# The Challenge of Treating Syringomyelia Associated with Extensive Arachnoiditis

**DOI:** 10.3390/jcm15197477

**Published:** 2026-09-25

**Authors:** Joseph Y. Choi, Carson Brantley, John D. Heiss

**Affiliations:** 1 Surgical Neurology Branch, National Institute of Neurological Disorders and Stroke, National Institutes of Health, Bethesda, MD 20892, USA; 2 School of Medicine, Wayne State University, Detroit, MI 48201, USA; 3 School of Medicine and Health Sciences, George Washington University, Washington, DC 20052, USA

**Keywords:** arachnoiditis, arachnoidopathy, syringomyelia, syrinx, diagnosis, treatment, prognosis

## Abstract

**Background/Objectives**: Extensive arachnoiditis associated with syringomyelia is a complex and multifactorial disease. This narrative review aims to synthesize current evidence on the terminology, pathogenesis, diagnostic evaluation, conservative and surgical management, and experimental approaches to arachnoiditis-related syringomyelia, with particular emphasis on the factors that limit durable treatment in extensive disease. **Methods**: PubMed was searched for English-language publications from January 2016 through August 2026 using the primary search terms “syringomyelia AND arachnoiditis,” supplemented by targeted searches for diagnostic, surgical, and experimental studies. Fifty-seven publications were included in the narrative synthesis. **Results**: Focal arachnoid pathology is generally more amenable to surgical reconstruction, whereas extensive multilevel arachnoiditis is associated with greater treatment complexity, recurrence, and reintervention. No single surgical strategy provides consistently durable control across all patterns of extensive disease, and bypass or diversion procedures may be required when direct restoration of the subarachnoid space is not feasible. Experimental studies further implicate cerebrospinal fluid obstruction, spinal cord tethering, altered intramedullary fluid handling, and persistent glial or fibrotic scarring in syrinx formation and treatment failure. **Conclusions**: The success of treatment for extensive arachnoiditis with syringomyelia depends on the extent of subarachnoid space involvement, particularly the number of spinal segments involved. Available surgical strategies remain limited by recurrent fibrosis, retethering, symptom recurrence, and treatment failure over time. Better treatment modalities are needed.

## 1. Introduction

Arachnoiditis is excess growth of fibrous tissue in the spinal subarachnoid space that is a consequence of inflammation within this space. The neurological symptoms and prognosis of these patients are variable and unpredictable [1,2]. Extensive arachnoiditis is a subtype of arachnoiditis that extends over more spinal segments than focal arachnoiditis [3]. In this review, extensive arachnoiditis involves 4 or more spinal cord segments. In extensive arachnoiditis, multilevel arachnoid scarring tethers the spinal cord, compartmentalizes the spinal subarachnoid space, and obstructs the cerebrospinal fluid (CSF) circulation [3]. Because these abnormalities extend across so many spinal levels, the disease may not be amenable to correction through a single localized decompression or reconstruction. After surgical treatment, recurrent fibrosis, spinal CSF obstruction, and syringomyelia are more likely to recur in extensive than focal arachnoiditis [3,4,5].

Various intrathecal inflammatory states can be followed by arachnoiditis. The nature of the inflammatory condition influences the anatomical location and scope of arachnoiditis. For example, thoracic arachnoidopathy is more often associated with trauma or spinal surgery, whereas cervical arachnoidopathy may be associated with meningitis-related inflammatory injury [5]. Regardless of the etiology of arachnoiditis, patients with extensive arachnoiditis typically have worse prognoses than those with focal arachnoiditis and the severity of spinal cord tethering, length of the spinal cord subarachnoid space affected, and extent of CSF flow obstruction are critical prognostic factors [3,4]. Magnetic resonance imaging (MRI) also detects syringomyelia, which can result from obstruction of the spinal CSF pathways by arachnoiditis [1,3]. Arachnoiditis-related syringomyelia frequently involves the thoracic cord and may extend into the cervical cord; in one contemporary series, all seven syrinxes involved thoracic levels, with five extending cervically [3]. Obstruction of subarachnoid CSF flow can create abnormal pressure gradients that promote interstitial fluid entry into the spinal cord and syrinx formation, and extensive adhesions may also disturb spinal cord venous circulation and impair interstitial fluid drainage [1,6].

Management of arachnoiditis-related syringomyelia involves making an initial decision regarding the feasibility of opening spinal CSF obstructions by removing, or creating a passage for CSF through, the intradural, extramedullary scarring within the spinal subarachnoid space [3,4]. In cases in which an obstruction to CSF flow is felt to be too extensive to resolve with a direct surgical approach on the spinal segments affected by arachnoiditis, a subarachnoid bypass procedure around the obstructed CSF pathways or direct syrinx drainage with syrinx shunts are options [7,8]. Syrinx shunting procedures require a myelotomy and placement of a tube within the syrinx, which adds an iatrogenic spinal cord injury to the syrinx-related myelopathy [7,8]. Syrinx shunts can also lead to long-term complications such as shunt dysfunction, dislodgement, and the need for reoperation, which further degrade long-term outcomes [4,7,9].

The lack of standardized terminology and treatment, incomplete understanding of syrinx pathogenesis, and limited durability of treatment for extensive disease prompted this review. This narrative review examines the terminology, etiologies, diagnostic evaluation, management decisions, surgical strategies, and translational approaches relevant to arachnoiditis-related syringomyelia with extensive arachnoiditis. It examines the factors that can reduce the durability of treatment with particular emphasis on why durable treatment remains challenging.

## 2. Materials and Methods

This study was conducted as a narrative review with a structured literature search. A narrative review was selected because the literature on arachnoiditis-related syringomyelia is heterogeneous, including case reports, case series, retrospective studies, reviews, and experimental studies examining diverse etiologies and treatment approaches.

PubMed was searched for English-language publications from January 2016 through August 2026. The primary search used the terms “syringomyelia AND arachnoiditis.” Additional targeted searches were performed for specific diagnostic, surgical, and experimental topics relevant to the review. Studies were included if they addressed the etiology, diagnosis, management, surgical treatment, outcomes, pathophysiology, or experimental modeling of spinal arachnoiditis and associated syringomyelia. Studies concerning related spinal arachnoid pathologies were considered when their findings were directly relevant to CSF obstruction, spinal cord tethering, or treatment of arachnoiditis-related syringomyelia.

Publications were not included when their subject matter was outside the scope of arachnoiditis-related syringomyelia or did not contribute information relevant to the objectives of this review. Publications were excluded if they were published before 2016, were not available in English, or lacked sufficient information to assess their relevance. Because of substantial heterogeneity in study populations, interventions, and reported outcomes, quantitative meta-analysis was not performed. The publication selection process is summarized in Figure 1.

## 3. Terminology, Classification, and Definition of Arachnoiditis-Related Syringomyelia

A major challenge in characterizing arachnoiditis is the lack of standardized terminology. Terms such as “adhesive arachnoiditis,” “arachnoid scarring,” “spinal adhesive arachnoidopathy,” “arachnoid fibrosis,” “arachnoid web,” and “arachnopathy” are often used inconsistently across the literature [5,10,11]. Palackdkharry et al. identified 63 disease names for related leptomeningeal inflammatory and fibrotic pathologies, highlighting the difficulty of comparing studies and developing uniform treatment algorithms [10]. In syringomyelia, it is clinically relevant to predict if relief of syringomyelia will be sufficient to arrest the myelopathy relevant to the arachnoid abnormality, or if spinal cord tethering must also be relieved [4,6].

Syringomyelia is a disease characterized by a fluid-filled cavity within the spinal cord [12]. In this review, “arachnoiditis-related syringomyelia” refers to a clinically relevant intramedullary cavity that develops in the setting of intradural arachnoid pathology capable of obstructing CSF flow, tethering the spinal cord, or both.

Arachnoiditis has traditionally been described as inflammation of the arachnoid membrane and subarachnoid space [1]. However, in chronic arachnoiditis, inflammatory findings may be limited, while adhesive scarring, fibrosis, and distortion or obliteration of the normal subarachnoid compartment predominate [13]. The term “adhesive arachnoiditis” emphasizes the mechanical consequences of bands and veils of scar tissue within the subarachnoid space that bridge from the arachnoid membrane to the spinal cord, binding them together and creating spinal cord tethering, CSF compartmentalization, syrinx formation, and progression [1,13]. More recently, “spinal adhesive arachnoidopathy” has been used as a broader term for adhesive intradural arachnoid disorders [5,11]. In this usage, arachnoiditis describes the inflammatory and adhesive disease process, whereas arachnoidopathy is a broader term that encompasses multiple structural arachnoid pathologies [5,11]. Zhang et al. explicitly adopted the term “spinal adhesive arachnoidopathy” to distinguish this broader group of disorders from adhesive arachnoiditis alone [5].

For this review, the distinction between focal and extensive arachnoiditis is central. Focal disease is confined to a short segment of the spinal canal, whereas extensive disease involves multilevel and often adhesive pathology. Published thresholds are inconsistent: Guillaumet et al. defined focal arachnoiditis as involvement of two spinal segments or fewer, whereas Bratelj et al. defined extensive arachnoiditis as involvement of more than three vertebral segments [4,14]. To provide a more uniform definition, this review uses “extensive arachnoiditis” pragmatically to describe adhesive arachnoidopathy across 4 or more spinal segments [14]. Multisegment disease of this length creates a subarachnoid obstruction that is challenging to open with intradural microsurgical approaches [3,4]. Several contemporary classification frameworks for spinal arachnoid pathology are summarized in Table 1.

Imaging is essential for diagnosis and classification of arachnoiditis, but MRI-based categories remain imperfect because imaging findings do not reliably distinguish active inflammation from mature fibrosis or consistently predict treatment response. El Homsi et al. revisited MRI classification of lumbar arachnoiditis and reported poor observer agreement, illustrating the difficulty of assigning patients to reproducible radiographic categories [15]. A multidimensional framework that includes etiology, morphology, anatomical location, longitudinal extent, effect on CSF flow, and clinical progression provides a more complete clinical picture of arachnoiditis-related syringomyelia than MRI alone. This multidimensional framework will be used to describe arachnoiditis-related syringomyelia throughout this review.

## 4. Etiology and Risk Factors for Arachnoiditis-Related Syringomyelia

The major etiologies and risk factors for arachnoiditis-related syringomyelia are summarized in Table 2. Infectious, inflammatory, traumatic, hemorrhagic, chemical, and iatrogenic insults can all initiate meningeal inflammation [2,5]. Different etiologies for arachnoiditis vary in their anatomical distribution, biological activity, and latency before adhesive disease becomes clinically apparent [5]. These distinctions are relevant when selecting treatment methods for extensive arachnoiditis consisting of postinfectious or posthemorrhagic fibrosis versus a focal arachnoid web or cyst [5,16,17].

Infection is an important cause of adhesive arachnoiditis. Bacterial, fungal, and viral meningitides may produce leptomeningeal inflammation, fibrosis, and obstruction of the spinal subarachnoid space [2,5]. Tuberculous meningitis is particularly prominent among reported infectious causes of spinal arachnoiditis, while fungal meningitis has also been associated with postinfectious spinal arachnoiditis [5,18]. In the systematic review by Zhang et al., meningitis accounted for approximately two-thirds of infection-associated cases, with tuberculosis representing the majority of reported bacterial meningitis cases [5]. The occurrence of arachnoiditis and syringomyelia in only a small proportion of patients with prior meningeal inflammation suggests that host factors also contribute to the propensity for inflammation to progress to arachnoiditis. In tuberculous meningitis, host-response variants involving the Toll/interleukin-1 receptor domain and leukotriene A4 hydrolase have been associated with individual differences in inflammatory intensity, suggesting that individual immune responses may influence the severity of meningeal injury [22]. Autoimmune and granulomatous disorders may also produce chronic meningeal inflammation. Neurosarcoidosis can involve the leptomeninges and spinal cord and should be considered when an active inflammatory cause of arachnoid disease is suspected [5,23]. Chemical and iatrogenic injury can produce an adhesive response within the spinal subarachnoid space similar to that from infection [1,5]. Intrathecal chemotherapy has been associated with neurological complications, including arachnoid inflammation [19]. Historical oil-based contrast agents, such as iophendylate (Pantopaque), may remain within the subarachnoid space and cause delayed fibrosis [20].

Intradural surgery is another important cause of arachnoiditis. Opening the dura and arachnoid disrupts the meninges, which maintain the blood–CSF barrier, and introduces blood products into the subarachnoid space around the meningeal opening [3]. The cut arachnoid membrane is sometimes impossible to close primarily, and secondary wound closure mechanisms reconstruct the arachnoid membrane and blood–CSF barrier. The presumed mechanism for intradural surgery causing postoperative CSF obstruction, fibrous adhesions, and tethering is that meningeal injury and subarachnoid blood products stimulate a repair response involving arachnoid cells and fibroblasts that can progress to excessive fibroconnective remodeling and scarring within the subarachnoid space [13,24].

Trauma and subarachnoid hemorrhage may also initiate arachnoid fibrosis. Following spinal cord injury, pericytes, fibroblasts, and growth factors participate in tissue repair that sometimes leads to formation of a fibrotic scar [5,21,25,26,27]. Blood products within the subarachnoid space may promote meningeal inflammation and subsequent adhesion formation. For example, a review by Jain et al. identified 18 previously reported cases of syringomyelia after aneurysmal subarachnoid hemorrhage, with latency between hemorrhage treatment and syringomyelia ranging from two weeks to 11 years [21].

Not all arachnoid pathology has an identifiable inflammatory or traumatic cause. Dorsal arachnoid webs, arachnoid cysts, and related focal lesions are considered idiopathic if there is no historical or radiographic evidence of infection, hemorrhage, trauma, or surgery [16]. These lesions can obstruct CSF flow and produce syringomyelia, as in arachnoiditis. However, idiopathic arachnoidopathies are often more anatomically localized than diffuse postinfectious or posthemorrhagic arachnoiditis and therefore more amenable to focal surgical correction [6].

Despite their etiologic diversity, these disorders converge on a common pathway of meningeal inflammation, fibrosis, spinal cord tethering, and disruption of CSF circulation. An overview of the prevention of arachnoiditis from this initiating insult is outlined in Figure 2. The likelihood of syrinx formation appears to depend on the initiating insult and the severity, location, circumferential extent, and duration of subarachnoid obstruction that results. Etiology should therefore be considered alongside anatomical extent when evaluating prognosis and determining whether the disease represents a focal surgically remediable lesion or a diffuse adhesive process.

## 5. Diagnostic Evaluation

Diagnostic evaluation should confirm arachnoid pathology, define its longitudinal extent, identify the location and severity of CSF obstruction, assess spinal cord tethering or compression, and determine whether a discrete anatomical target exists.

Clinical evaluation remains essential because treatment decisions are driven by neurological progression. Assessment should document myelopathy, radiculopathy, gait dysfunction, weakness, sensory loss, neuropathic pain, spasticity, and bowel or bladder impairment. Prior meningitis, spinal trauma, subarachnoid hemorrhage, intradural surgery, spinal anesthesia, intrathecal medications, and prior myelography should be specifically reviewed because they may suggest the etiology and expected distribution of arachnoiditis [2,5,28]. Serial examinations are necessary to distinguish stable, longstanding deficits from progressive neurological deterioration [5,29].

MRI is the primary imaging modality for evaluating arachnoiditis-related syringomyelia. Standard evaluation should include MRI of the relevant spinal region, and often the entire craniospinal axis, to define syrinx length, maximal diameter, cord edema, cord atrophy, spinal cord displacement, and possible associated cranial, craniocervical, or spinal pathology. Reported MRI findings of adhesive arachnoiditis include nerve root clumping, peripheral adherence of nerve roots to the thecal sac, an “empty thecal sac” appearance, intrathecal soft-tissue filling defects, loculated CSF collections, arachnoid septations, cord tethering, syrinx formation, cord swelling with T2 hyperintensity, cord atrophy, and intrathecal calcifications [1,15,28,34]. Reports should specify the longitudinal extent of disease; whether adhesions are dorsal, ventral, circumferential, or multilevel; and whether discrete levels of CSF obstruction are present.

Contrast-enhanced MRI may identify active inflammatory, infectious, neoplastic, or postoperative meningeal disease and help exclude alternative causes of syringomyelia, including intramedullary tumors, inflammatory myelitis, infection, and leptomeningeal malignancy [2,29]. Meningeal enhancement may indicate inflammation or blood–CSF barrier disruption, but its absence does not exclude chronic adhesive arachnoiditis, which may be dominated by mature fibrosis.

Advanced MRI techniques may improve surgical planning by clarifying the anatomy and physiology of CSF obstruction. Cardiac-gated phase-contrast cine MRI can evaluate CSF flow at the foramen magnum and within the spinal canal and may help identify physiologic obstruction that is not obvious on static imaging [35]. Heavily T2-weighted sequences, including fast imaging employing steady-state acquisition and constructive interference in steady-state imaging, may improve visualization of arachnoid membranes, adhesions, septations, cyst walls, and communications between the syrinx and surrounding CSF spaces [3,36]. Time-spatial labeling inversion pulse MRI and three-dimensional constructive interference in steady-state imaging have also been described as useful adjuncts for distinguishing arachnoid cysts and other intradural arachnoid abnormalities from surrounding CSF [5]. Together, these techniques may clarify whether a discrete, focal obstruction exists within otherwise extensive adhesive disease.

Special attention is required when syringomyelia is located near the craniocervical junction because foramen magnum arachnoiditis can mimic Chiari malformation. Hatano et al. reported that a shorter fourth ventricle-to-syrinx distance was characteristic of syringomyelia associated with foramen magnum arachnoiditis, with a cutoff of less than 11 mm showing high sensitivity and specificity for differentiating foramen magnum arachnoiditis from Chiari malformation [37]. Distinguishing these disorders is important because their underlying anatomical abnormalities and operative objectives differ.

Computed tomography (CT) myelography remains useful when MRI is limited by artifact, when the level of CSF obstruction remains uncertain, or when a suspected block is not adequately characterized by MRI. CT may also demonstrate arachnoid calcification or ossification [38]. The historical oil-based contrast agents that were associated with adhesive arachnoiditis are no longer used. MRI has replaced myelography as the first-line study, and CT myelography should be reserved for cases in which the additional anatomical information is likely to change management [5,39].

Lumbar puncture is not required for every patient with chronic adhesive arachnoiditis but may be appropriate when infection, inflammatory meningitis, malignancy, or another active inflammatory disorder is suspected (Figure 2). Evaluation may include cell count, differential, protein, glucose, culture, cytology, and disease-specific testing. Pleocytosis may support active inflammatory disease. Elevated protein is seen with blockage of the spinal CSF pathways and normal CSF findings do not exclude chronic post-inflammatory arachnoiditis. Postoperative or traumatic blood products may also produce nonspecific inflammatory abnormalities in the CSF. CSF findings should therefore be interpreted in relation to timing from previous surgery or trauma, imaging, symptoms, and the suspected etiology of arachnoiditis [2,13,29].

When preoperative imaging does not fully define the functional significance of adhesions, intraoperative ultrasound may assess syrinx morphology, cord pulsation, adhesions within the subarachnoid space, and the response to decompression. Direct measurement of pressure gradients across obstructed spinal segments has also been explored to confirm physiologically significant CSF blockage and guide bypass or diversion strategies [8]. These approaches remain adjunctive, but they reflect an important principle: in extensive arachnoiditis, the existence of adhesions is known, but the question is whether restoring or bypassing a specific CSF pathway is feasible.

No single diagnostic test fully characterizes arachnoiditis-related syringomyelia. Evaluation should integrate neurological progression, disease etiology, longitudinal extent of arachnoiditis and the syrinx, morphology, CSF-flow physiology, and evidence of spinal cord injury. The practical objective is to determine whether symptoms are attributable to a defined obstruction or tethering point and whether the available anatomical information is sufficient to guide management.

## 6. Indications for Surgery vs. Conservative Management

The decision to operate considers the anatomical extent of intramedullary syrinx and the extramedullary adhesive process, and the likelihood of permanent surgical restoration of the spinal subarachnoid space, spinal cord untethering, and syrinx resolution. Surgery should not be based on syrinx size alone. Management should instead consider neurological progression, symptom burden, disease extent, spinal cord tethering, the location and severity of CSF obstruction, and whether intervention can restore or bypass CSF pathways durably (Figure 3) [4,40].

Conservative management is appropriate for patients with stable neurological status, mild symptoms, or imaging findings that are not clearly associated with progressive myelopathy. In a systematic review of post-traumatic syringomyelia, 198 patients were managed conservatively; 88% remained neurologically stable, 10% deteriorated, and 2% improved [40]. These findings support observation in selected patients with stable symptoms, longstanding deficits, or no discrete surgically remediable obstruction. Observation should include serial neurological examinations, interval MRI when clinically indicated, symptomatic treatment, and rehabilitation [41].

Symptomatic treatment remains an important component of conservative care even though it does not reverse the underlying adhesive pathology. Neuropathic pain and spasticity may be managed with standard pharmacological therapies tailored to symptom severity and treatment tolerance [41]. Physical and occupational therapy may help maintain mobility, improve function, and reduce disability, although high-quality evidence for active conservative management in primary spinal syringomyelia remains limited [41].

Surgery is generally considered for documented neurological deterioration, worsening myelopathy or radiculopathy, clinically correlated syrinx enlargement, disabling symptoms attributable to tethering or CSF obstruction, or a focal lesion likely to be surgically correctable [14,42]. The clearest candidates are patients with a localized arachnoid web, cyst, adhesive band, or short-segment obstruction that provides a discrete anatomical target. In contrast, patients with extensive arachnoiditis require a more cautious threshold for surgery because multilevel adhesive disease is less likely to be fully corrected by localized decompression and is associated with higher rates of recurrence and reintervention [4,6].

Intradural surgery may reduce syrinx size, improve CSF flow, or stabilize neurological decline, but it may also introduce blood products, inflammation, and additional scarring into an already compromised subarachnoid space. In extensive disease, the realistic goal is often stabilization rather than neurological recovery, particularly when deficits reflect chronic spinal cord injury. Patients should therefore understand that syrinx reduction may not reverse longstanding neurological deficits and that the primary objective may be stabilization rather than recovery [6,14].

The presence of an active infectious, inflammatory, autoimmune, neoplastic, or other treatable process should also influence management. Treatment of the underlying disorder should precede or accompany surgical intervention when appropriate. Established fibroadhesive disease, however, cannot be reversed by medical therapy alone [29].

Management should therefore proceed from clinical trajectory to anatomical feasibility: stable patients may be observed, whereas progressive patients should be evaluated for a discrete obstruction or tethering point that can be treated with an acceptable risk of morbidity. When surgery is indicated, the operative strategy should be selected according to the location and extent of the adhesive process and the probability of restoring the patency of the subarachnoid space and eliminating spinal cord tethering. CSF diversion procedures, if effective, restore CSF flow around or through an obstruction in the subarachnoid space but do not correct spinal cord tethering.

## 7. Surgical Treatment Options

Surgical procedures for syringomyelia associated with arachnoiditis can act through restoring or bypassing obstructed CSF pathways, untethering the spinal cord, reducing syrinx pressure, or diverting CSF to an absorptive compartment [2]. Procedure selection depends on the location and extent of adhesive disease, the presence of spinal cord tethering, and the availability of a patent CSF compartment. Focal lesions may be amenable to reconstruction of the subarachnoid space, whereas extensive disease may require diversion, bypass, or combined approaches [3]. An outline of these interventions and their indications is provided in Table 3.

### 7.1. Arachnolysis (Microsurgical Lysis of Adhesions) with Duraplasty

Microsurgical arachnolysis with or without duraplasty is the principal reconstructive operation for focal arachnoiditis. Through laminectomy or laminoplasty, the dura is opened, adhesions are divided, and the spinal cord is untethered. If necessary, an expansile duraplasty is performed to enlarge the subarachnoid space and restore CSF flow [4]. Recent series of focal spinal arachnoid webs have reported favorable clinical and radiographic outcomes after laminectomy or laminoplasty with intradural arachnoid lysis or web excision [16,17].

The effectiveness of arachnolysis decreases as the disease becomes more extensive [4]. Long-segment adhesions may extend beyond the operative exposure, and surgical manipulation may introduce blood products and inflammation into a subarachnoid space already prone to scarring. Arachnolysis with duraplasty is therefore best suited to focal webs, cysts, or focal arachnoiditis involving less than 3 vertebral levels [3]. In extensive disease, limited arachnolysis can release a dominant tethering point or facilitate an adjunctive bypass or shunt.

### 7.2. Shunting Procedures

Shunting is considered when reconstruction of the subarachnoid space is not feasible or when syringomyelia persists after restoration of CSF flow has been attempted. Traditional syrinx-directed procedures include syringosubarachnoid, syringoperitoneal, and syringopleural shunts. Although these operations can decompress the syrinx, they require myelotomy and catheter placement within the spinal cord, creating risks of neurological injury, obstruction, infection, disconnection, and delayed failure [2,9]. Syringosubarachnoid shunts may also fail when the distal catheter drains into a scarred or poorly communicating subarachnoid compartment.

Modified shunting strategies attempt to place the distal catheter beyond the diseased compartment of the spinal subarachnoid space or into a more reliable absorptive space outside the spine. Naito et al. used a programmable syringoperitoneal shunt in 15 patients with extensive spinal adhesive arachnoiditis, permitting postoperative adjustment of drainage [7]. Cho et al. described a syringo-cisterna magna shunt for cervical postoperative arachnoiditis, thereby avoiding drainage into a scarred spinal subarachnoid compartment [43]. Syrinx-to-subarachnoid shunting may also be effective in selected patients, but its durability depends on the patency of the receiving CSF space [9].

### 7.3. Subarachnoid Bypass Procedures

Subarachnoid bypass procedures reestablish CSF continuity without directly entering the syrinx. These procedures route CSF around an obstructed subarachnoid segment, addressing the extramedullary pressure gradient while avoiding myelotomy.

Recent series have refined this approach. Yuan et al. described a modified subarachnoid-subarachnoid bypass for posttraumatic syringomyelia, and Zhang et al. applied a similar minimally invasive strategy to postoperative adhesive syringomyelia [8,44]. Akgun et al. also reported long-segment subarachnoid–subarachnoid shunting in patients with recurrent post-traumatic or postinfectious arachnoid pathology [25]. Subarachnoid–subarachnoid bypass requires patent subarachnoid spaces above and below the obstructed segment [8]. Diffuse multilevel disease that provides no reliable proximal or distal receiving compartment is a contraindication for subarachnoid–subarachnoid bypass [45].

### 7.4. Thecoperitoneal Shunting

Thecoperitoneal shunting diverts CSF from the thecal sac to the peritoneal cavity instead of draining the syrinx directly. This strategy avoids myelotomy and is intended to bypass impaired spinal CSF drainage or absorption. Yuan et al. subsequently reported thecal–peritoneal shunting for extensive spinal adhesive arachnoiditis or lumbosacral outlet obstruction with syringomyelia. In this prospective series of 20 patients, CSF was diverted to the peritoneal cavity to reduce pathological pressure gradients associated with impaired lumbosacral drainage [45]. Because the procedure does not require reconstruction of the entire scarred subarachnoid space, it may be particularly applicable to extensive disease. Longer follow-up is needed to define the treatment’s durability, rate of shunt dependency, and appropriate patient selection.

### 7.5. Posterior Fossa Decompression

Posterior fossa decompression is considered when syringomyelia is associated with arachnoiditis at the craniocervical junction or foramen magnum. The surgical procedure must restore communication among the fourth ventricle, cisterna magna, and spinal subarachnoid space and decompress an associated Chiari I malformation, if present. Depending on the anatomy, treatment may include decompression, adhesiolysis, opening of the fourth ventricular outlets, duraplasty, or fourth ventricle-to-subarachnoid shunting [46].

Davidoff et al. reported that most syrinx cavities in patients with craniocervical junction arachnoiditis communicated with the fourth ventricle [46]. Posterior fossa decompression with fourth ventricle-to-spinal subarachnoid shunting was considered a reasonable strategy in this anatomical subtype, although recurrence requiring reoperation remained a concern.

### 7.6. Flexible Thecoscopy (Endoscopic Approaches)

Flexible thecoscopy is a novel technique used to treat patients with chronic adhesive arachnoiditis. Its use has been proposed as an adjunct to laminectomy and arachnolysis for patients with extensive arachnoiditis extending over 3 spinal segments. The flexible neuroendoscope is introduced into the surgical procedure after open microsurgical techniques have treated intrathecal areas with dense arachnoiditis. The flexible neuroendoscope is advanced into the spinal subarachnoid space superiorly and inferiorly to the laminectomy until the subarachnoid space communicates with circulating CSF. This minimally invasive management of scarring in the spinal subarachnoid space allows operative exposures involving fewer spinal cord segments than an approach requiring laminectomy over more than 3 spinal segments [3]. As such, less invasive strategies such as thecoscopy may reduce the extent of open surgical exposure in patients with arachnoiditis-related syringomyelia.

Mastorakos et al. evaluated flexible thecoscopy in patients with extensive spinal arachnoiditis. Among seven patients with associated syringomyelia, five demonstrated radiographic syrinx reduction. The mean maximal syrinx diameter decreased by 3.1 mm [3].

These early findings suggest that flexible thecoscopy may extend adhesiolysis beyond the margins of a limited open exposure while avoiding multilevel laminectomies. However, the available evidence consists of small, selected series, and radiographic syrinx reduction does not establish durable neurological benefit. The relative role of thecoscopy compared with open arachnolysis, subarachnoid bypass, and shunting therefore remains uncertain.

## 8. The Central Challenge: Extensive Arachnoiditis

The central therapeutic problem in extensive arachnoiditis is not the absence of operative options, but the inability of any single procedure to provide consistently durable control across heterogeneous patterns of multilevel disease. In a series of 19 patients with syringomyelia associated with spinal arachnoiditis, Koyanagi et al. reported 13 patients with secondary disease related to conditions such as prior spinal surgery or hemorrhage. Among these patients, three developed motor deterioration after arachnoid lysis, six required eight additional operations, and only four showed improvement in neurological grade over a mean follow-up of 44.3 months [6]. These findings illustrate that initial surgical treatment may not provide durable disease control in patients with extensive secondary adhesive pathology.

Long-term outcomes have varied considerably. A recent systematic review of post-traumatic syringomyelia included 68 studies and 1175 patients and found substantial variability in postoperative outcomes, with reported overall improvement ranging from 58.0% to 93.5% across longitudinal and before–after studies [47]. Other post-traumatic cohorts have reported more favorable results in selected patients. Bratelj et al. reported improvement in neurological deficits in 65.9% of surgically treated patients and improvement in spasticity or neuropathic pain in 50.0%. However, 37.3% required revision surgery, and revision was more common in patients with extensive than focal arachnoiditis (43.2% vs. 16.7%) [14]. Holmström et al. similarly reported favorable outcomes and high patient satisfaction after untethering with or without syrinx drainage [48].

These findings demonstrate that selected patients can benefit from intervention, but they also highlight the heterogeneity of the available evidence. Most larger surgical cohorts treat post-traumatic tethering, which may differ in distribution, tissue biology, and surgical accessibility from diffuse postinfectious or inflammatory arachnoiditis. Their outcomes may therefore not be directly applicable to patients with circumferential fibrosis, multiple noncommunicating CSF compartments, or disease extending across much of the spinal canal. Even within extensive disease, a dominant focal block, diffuse circumferential fibrosis, and several discontinuous adhesive segments may require different strategies and carry different prognoses. The difficulty of achieving durable untethering is also evident among patients requiring revision surgery. Capone et al. evaluated 25 patients who underwent at least two operations for post-traumatic spinal cord tethering and syringomyelia and found that the location and extent of tethering were associated with recurrent symptomatic disease requiring further intervention [49].

Results from modified bypass and diversion procedures have also been inconsistent. Akgun et al. reported neurological improvement without recurrence over 4–66 months after adhesiolysis and long-segment subarachnoid–subarachnoid shunting in patients with post-traumatic or postinfectious disease [25]. Conversely, Kin et al. described early recurrence after syringo-subarachnoid and subarachnoid–subarachnoid shunting in a patient with treatment-resistant extensive arachnoiditis, followed by a more durable response to syringoperitoneal diversion [50]. These contrasting experiences emphasize that treatment must be individualized according to disease etiology, the distribution of adhesions, the availability of patent CSF compartments, and prior operations.

The current literature does not establish one preferred surgical strategy for extensive arachnoiditis. Published evidence consists largely of retrospective cohorts, small case series, and technically selected populations with different etiologies, anatomical patterns, procedures, and outcome definitions. Recent systematic reviews similarly demonstrate substantial heterogeneity in operative techniques, study populations, and reported outcomes [40,47]. Until stronger comparative evidence is available, the principal challenge remains selecting the procedure most likely to preserve function while minimizing the cumulative risks of repeated intradural intervention. The limited ability of current clinical evidence to predict durability also underscores the need for experimental systems that reproduce multilevel fibrosis and postoperative re-adhesion.

## 9. Experimental Models and Translational Insights

Experimental models have clarified mechanisms of syrinx formation, but most reproduce focal subarachnoid obstruction rather than the chronic, multilevel adhesive disease that makes extensive arachnoiditis difficult to treat. Contemporary models have examined CSF obstruction, post-traumatic cavity formation, perivascular fluid transport, local osmoregulation, glial scarring, and regenerative therapies (Table 4) [51,52,53,54,55].

Recent experimental models support a multifactorial model of syrinx formation in which abnormalities of CSF circulation interact with spinal cord injury and altered intramedullary fluid handling. Ma et al. produced syringomyelia by chronically obstructing spinal CSF flow through extradural compression in rats, supporting an association between sustained CSF-flow disturbance and cavity formation [53]. Xu et al. developed a post-traumatic model combining contusive spinal cord injury with subarachnoid blood injection, producing spinal cord tethering and chronically expanding extracanalicular cysts [55].

More recent studies suggest that abnormal fluid transport within the spinal cord may also contribute to syrinx progression. Johnson et al. found enlargement of arteriolar perivascular spaces specifically within the perisyringeal spinal cord in a rat model of post-traumatic syringomyelia [52]. Pukale et al. demonstrated that fluid surrounding the syrinx was more hypertonic than CSF and identified regional upregulation of aquaporins, osmolytes, and related transport pathways, suggesting that local osmotic dysregulation may contribute to fluid accumulation within the spinal cord [54].

Experimental evidence also suggests that restoring CSF circulation may not be sufficient to reverse an established syrinx once secondary spinal cord changes have developed. Cui et al. used a rat syringomyelia model in which decompression restored subarachnoid CSF flow, yet some syringes failed to improve. Persistent syringes were associated with extensive perisyringeal glial scarring, and pharmacological inhibition of astrocytic scar formation promoted syrinx reduction and improved neuronal, axonal, and functional recovery [51].

MRI remains useful for longitudinal assessment of experimental syringomyelia, allowing for serial evaluation of syrinx formation and response to intervention. Contemporary rat models have used serial MRI to document development of syringomyelia after chronic CSF obstruction and to assess syrinx response following decompression or experimental therapy [51,53]. However, radiographic reduction of an experimental syrinx does not establish that a treatment can maintain long-term CSF pathway patency or prevent recurrent adhesions after surgery.

Experimental studies have also begun to evaluate therapies directed at the consequences of syrinx formation. Xu et al. reported that transplantation of human neural precursor or induced pluripotent stem cell-derived neuroepithelial-like cells reduced continued cavity expansion in a rat model of post-traumatic syringomyelia [55]. Subsequent work using induced pluripotent stem cell-derived neural stem cells demonstrated reductions in cyst volume, glial scarring, inflammatory cell activation, and neurite-inhibitory extracellular matrix components, together with evidence of axonal repair [56]. Although these approaches suggest that regenerative therapies may limit secondary spinal cord injury, they do not correct the underlying adhesive obstruction.

Durable treatment of extensive arachnoiditis will likely require strategies that address both the intramedullary injury and the extramedullary fibrotic process that disrupts CSF circulation and creates spinal cord tethering. The principal limitation preventing translation of these animal model findings to human extensive arachnoiditis is that current models rarely reproduce extensive arachnoiditis as described in this review. Experimental insults generally produce relatively focal pathology over short observation periods, whereas patients with extensive arachnoiditis may have circumferential fibrosis spanning multiple levels, several noncommunicating CSF compartments, prior intradural operations, and recurrent postoperative scarring. Models using kaolin, epidural compression, subarachnoid blood, or traumatic spinal cord injury reproduce selected components of this disease but not the complete chronic fibroadhesive phenotype of human extensive arachnoiditis [51,54,55]. More clinically representative models should therefore incorporate multilevel obstruction, spinal cord tethering, and re-adhesion after surgical treatment. Such systems would provide a stronger platform for evaluating therapies intended not only to reduce syrinx size, but also to restore and preserve CSF circulation and resolve spinal cord tethering in the setting of extensive adhesive disease.

## 10. Discussion

Arachnoiditis-related syringomyelia remains difficult to treat successfully because the underlying disorder is a diffuse, heterogeneous, and potentially recurrent scar-forming process. Current operations can untether the spinal cord, reopen or bypass obstructed CSF pathways, and decompress the syrinx, but none consistently provides durable control across all patterns of extensive disease. Future progress will depend on stratifying patients according to disease etiology, anatomical extent, CSF physiology, biological activity, prior operations, and the likelihood of recurrent fibrosis.

A major unresolved question is whether arachnoiditis can be staged according to its biological activity. No pharmacological therapy has been shown to reverse the established fibroconnective adhesions that characterize chronic extensive arachnoiditis. In contrast, active inflammatory disease may respond to treatment of an underlying infectious, autoimmune, or other inflammatory condition [29]. Panackal et al. identified elevated CSF inflammatory cell populations, soluble immune markers, and neurofilament light chain in patients who developed spinal arachnoiditis after cryptococcal meningoencephalitis, demonstrating that measurable inflammatory phenotypes exist in some patients [18]. Conversely, Choi et al. found low preoperative CSF white cell counts and a predominantly fibroblast-associated, scar-forming tissue phenotype in 60 patients undergoing surgery for arachnoiditis-related syringomyelia, supporting the presence of a more fibrotic phenotype in established surgical disease [13]. Prospective studies should evaluate whether combinations of CSF biomarkers, meningeal enhancement, CSF-flow measurements, and operative tissue characteristics can distinguish ongoing inflammation from mature fibrosis and predict progression, surgical response, or recurrence.

Preventing postoperative re-adhesion is another central priority. Walker et al. described the placement of human amniotic membrane after intradural adhesiolysis in 14 patients, providing preliminary evidence that a biological anti-adhesion barrier can be applied safely during spinal detethering procedures [57]. Recent molecular studies also suggest that biological prevention of recurrent fibrosis may eventually complement mechanical anti-adhesion barriers. Single-nucleus transcriptomic analysis of human adhesive arachnoid tissue identified fibroblast populations enriched for extracellular matrix genes, including COL1A1, COL3A1, and FN1, and identified additional molecular pathways that may represent future therapeutic targets [24]. These approaches remain investigational, but they suggest that future surgery may combine mechanical restoration of CSF pathways with biomaterials designed to separate injured surfaces, reduce inflammatory signaling, or locally deliver therapeutic agents.

Pharmacological control of arachnoid fibrosis remains less developed. Hackert et al. reported no improvement after corticosteroids, methotrexate, or plasmapheresis in three patients with longstanding chronic adhesive arachnoiditis, whereas one patient treated during the early inflammatory stage recovered completely [29]. More recently, transcriptomic profiling of adhesive arachnoiditis identified fibroblast-associated extracellular matrix pathways and potential molecular targets, including cathepsin S and nicotinamide phosphoribosyl transferase, although these findings have not yet been translated into established antifibrotic therapy [24]. Such strategies may ultimately be most useful before dense multilevel adhesions form or as perioperative adjuncts intended to reduce recurrent fibrosis rather than reverse mature scar tissue.

The evidence base for extensive arachnoiditis also requires substantial methodological improvement. Extensive arachnoiditis is uncommon, and published studies include different etiologies, anatomical distributions, operations, follow-up durations, and definitions of success. This limitation is evident in contemporary reviews: a 2024 systematic review of spinal adhesive arachnoidopathy identified 147 clinical studies, of which 136 were case reports or case series and only 11 were retrospective studies [5]. Similarly, a 2026 systematic review of post-traumatic syringomyelia included 37 longitudinal or before–after studies and 31 case reports and found wide variation in reported postoperative improvement [47]. Prospective multicenter registries should use standardized definitions of disease extent and report neurological function, pain, spasticity, mobility, quality of life, syrinx progression, treatment failure, and reintervention over long-term follow-up. Radiographic change should be reported alongside patient-centered and neurological outcomes.

Previous reviews have addressed postinfectious arachnoiditis with syringomyelia, spinal adhesive arachnoiditis more broadly, or the wider spectrum of spinal adhesive arachnoidopathy [1,2,5]. The present review differs by focusing specifically on extensive arachnoiditis-related syringomyelia and integrating disease extent with diagnostic evaluation, surgical feasibility, contemporary bypass and diversion procedures, and experimental models. Its principal advantage is this focused, anatomy-based synthesis of a particularly challenging clinical subgroup. Its principal limitation is that the available evidence remains heterogeneous and is dominated by retrospective cohorts and small case series, limiting direct comparisons among surgical procedures and conclusions regarding long-term durability.

Improving treatment will require integration of four complementary strategies: accurate anatomical characterization of CSF obstruction, accurate anatomical and physiological characterization of spinal cord tethering, selection of the most appropriate reconstructive or diversion procedure, and biological prevention of recurrent inflammation and fibrosis. Complementary experimental models must reproduce multilevel fibrosis and postoperative re-adhesion. Until these advances are achieved, treatment should remain individualized, with realistic goals of preserving neurological function, reducing symptom burden, limiting repeated intradural intervention, and achieving the most durable CSF pathway restoration possible.

## 11. Conclusions

Arachnoiditis-related syringomyelia is particularly difficult to treat when arachnoiditis is extensive because multilevel fibrosis, spinal cord tethering, CSF compartmentalization, and recurrent scarring limit durable restoration of CSF pathways. Focal disease is more amenable to reconstructive surgery, whereas extensive disease often requires individualized bypass or diversion strategies with realistic goals of neurological stabilization rather than complete recovery. Future progress will depend on improved patient stratification, standardized long-term outcomes, and strategies that prevent recurrent fibrosis while preserving CSF circulation.

## Figures and Tables

**Figure 1:**
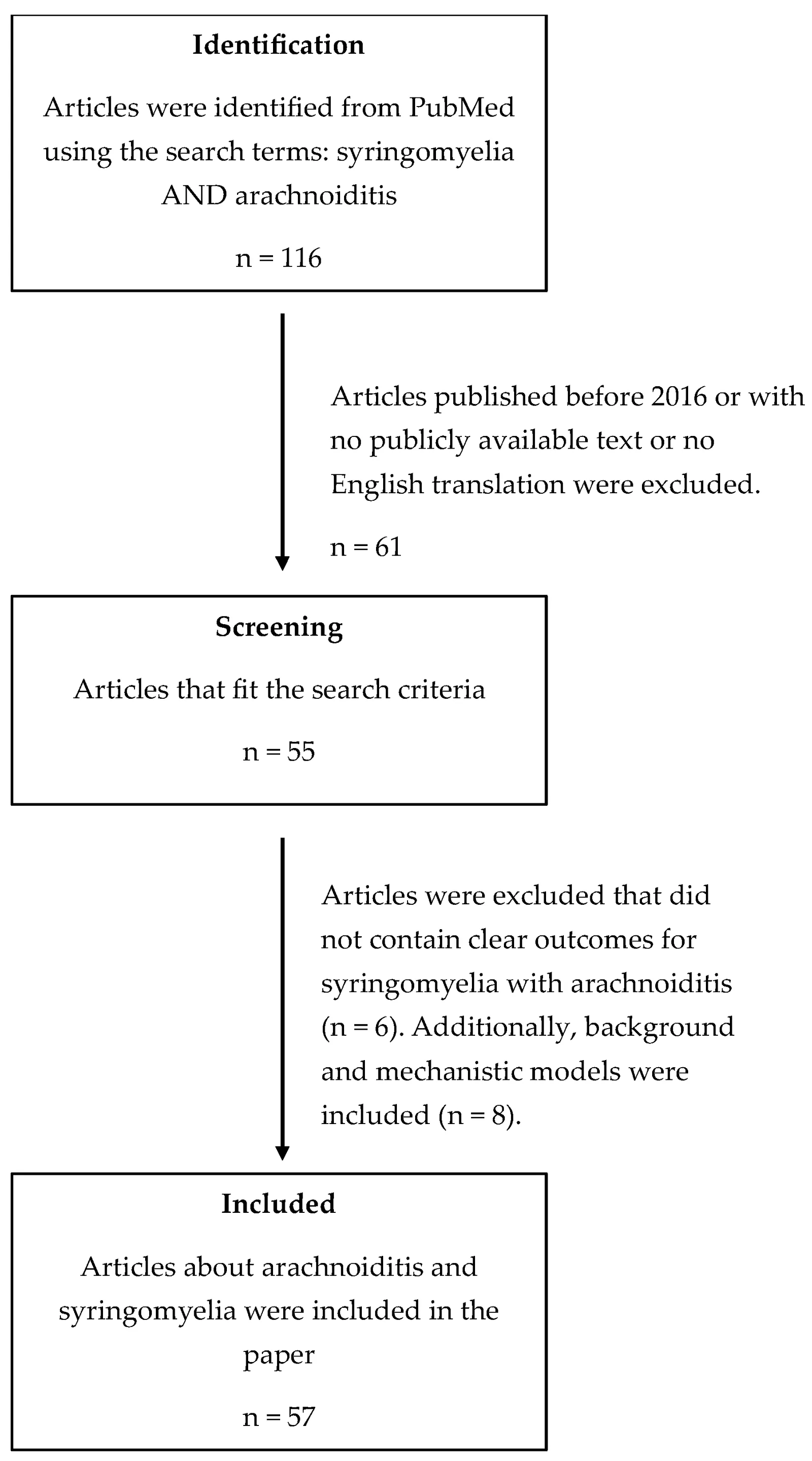
Article Screening.

**Figure 2:**
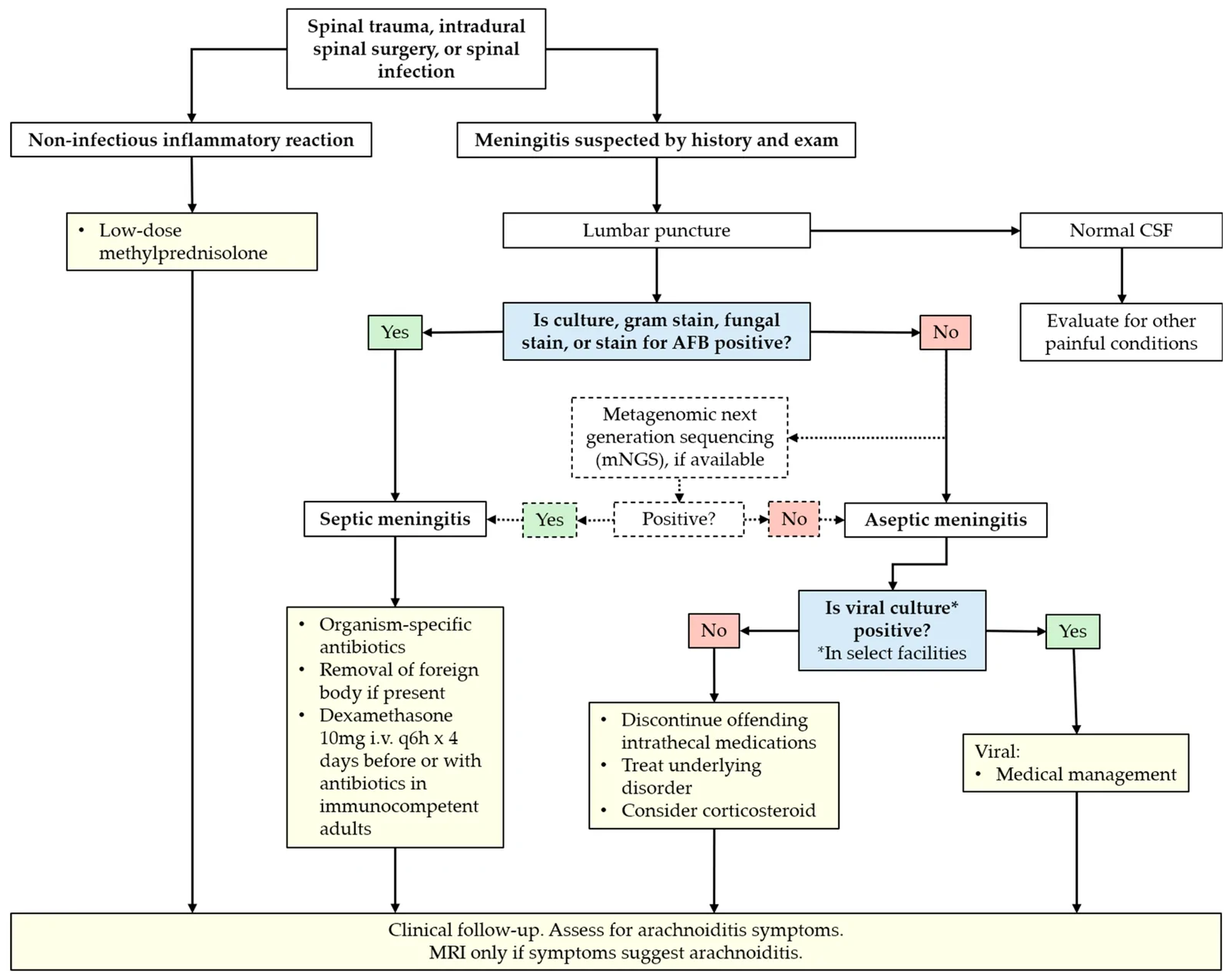
Conceptual framework for the evaluation and early management of conditions that may precede arachnoiditis [2,5,28,29,30,31,32,33]. Note: AFB, acid-fast bacilli; CSF, cerebrospinal fluid; i.v., intravenous; mNGS, metagenomic next-generation sequencing; MRI, magnetic resonance imaging; q6h, every 6 h.

**Figure 3:**
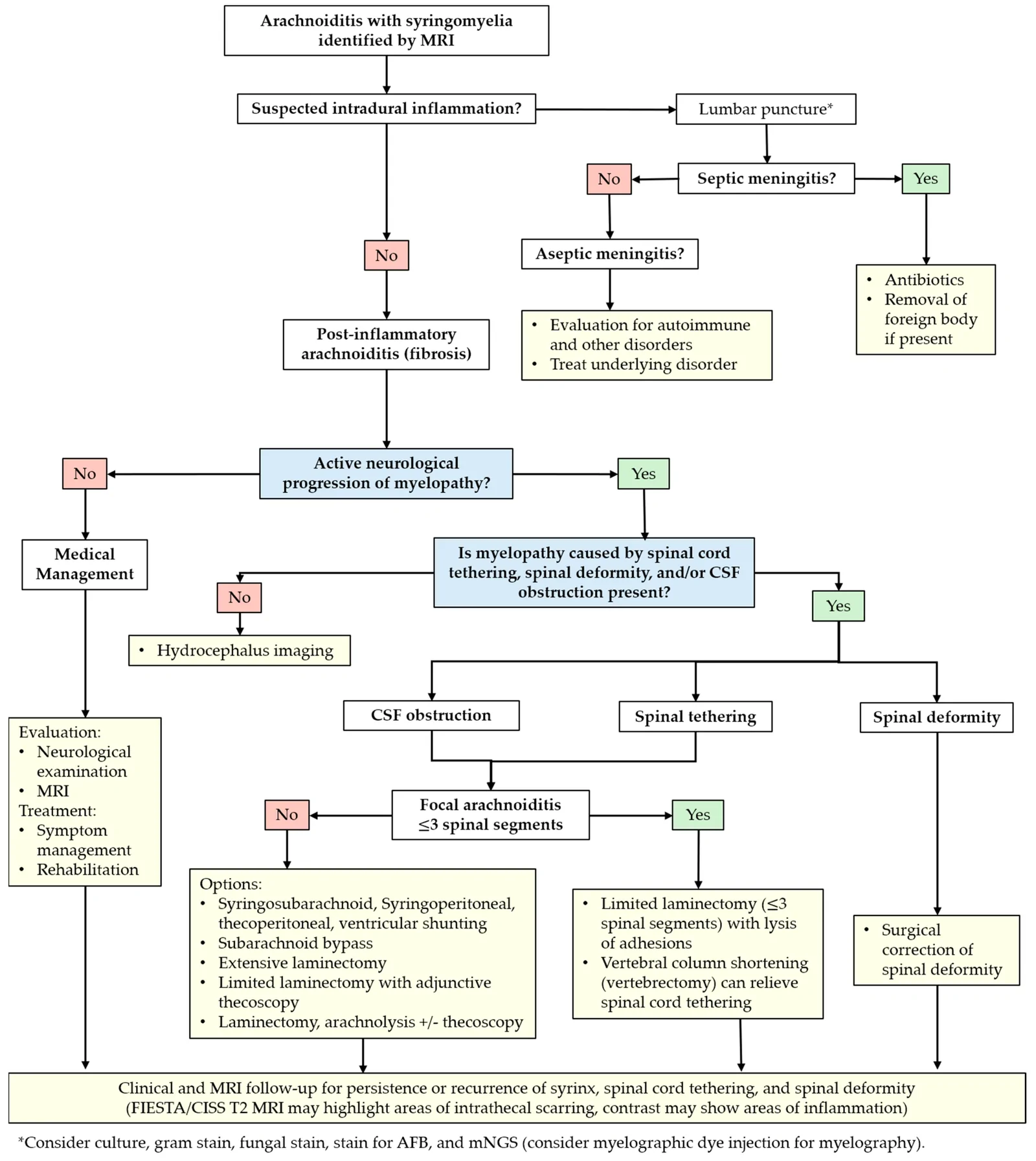
Diagnostic and management framework. Note: AFB, acid-fast bacilli; CISS, constructive interference in steady state; CSF, cerebrospinal fluid; FIESTA, fast imaging employing steady-state acquisition; mNGS, metagenomic next-generation sequencing; MRI, magnetic resonance imaging; T2, T2-weighted imaging.

**Table 1:** Contemporary classification frameworks relevant to arachnoiditis-related syringomyelia.

Author, Year	Classification/Framework	Reference
El Homsi et al., 2021	MRI-based classification of lumbosacral arachnoiditis using four imaging-pattern groups, including a fourth “nonspecified” group for indeterminate findings	[15]
Guillaumet et al., 2021	Classified arachnoiditis associated with nonforaminal syringomyelia by longitudinal extent; focal disease ≤ 2 spinal segments, with more extensive disease analyzed separately	[4]
Bratelj et al., 2023	Defined extensive arachnoiditis as involvement of >3 vertebral segments, demonstrating greater revision rates in extensive than focal disease	[14]
Zhang et al., 2024	Classified spinal adhesive arachnoidopathy into adhesive arachnoiditis, arachnoiditis with arachnoid cyst, arachnoid calcification/scarring, and arachnoid web/fibrosis	[5]
Venard et al., 2026	Integrated spinal meningeal classification separating dural disorders from arachnoid disorders, with arachnoid pathologies categorized as cysts, webs, and arachnoiditis	[11]

**Table 2:** Etiology and risk factors for arachnoiditis-related syringomyelia [2,5,16,17,18,19,20,21].

Etiologic Category	Representative Causes	Typical Latency	Expected Disease Pattern	Clinical or Treatment Implications
Post-Infectious & Post-Inflammatory Arachnoiditis	Tuberculosis, fungal meningitis, sarcoidosis	Months to years	Often diffuse or multilevel	Assess for active inflammation; extensive fibrosis may remain after infection resolves
Post-Traumatic Arachnoiditis	Spinal cord injury	Delayed, often years	Focal tethering or multilevel scarring	Untethering may benefit selected patients with focal arachnoiditis
Post-Hemorrhagic Arachnoiditis	Subarachnoid hemorrhage	Approximately 1–11 years in reports	Diffuse blood product-associated adhesions	Delayed progression; presyrinx may precede syrinx
Post-Surgical Arachnoiditis	Intradural surgery	Variable	Localized or multilevel postoperative adhesions	Risk of retethering and recurrent fibrosis
Post-Chemical (Iatrogenic) Arachnoiditis	Iophendylate (Pantopaque), intrathecal chemotherapy, spinal anesthesia	Sometimes decades	Variable, potentially diffuse	Exposure history may provide the diagnosis
Idiopathic Arachnoidopathy	Dorsal arachnoid web or cyst	Variable	Usually focal	Provides a discrete and remediable surgical target

**Table 3:** Surgical treatment options for arachnoiditis-related syringomyelia [2,3,4,7,8,9,43,44,45,46].

Procedure	Primary Objective	Most Appropriate Anatomy	Principal Advantages	Major Limitations
Laminectomy and arachnolysis, with or without duraplasty	Restore native CSF flow and untether cord	Focal web, cyst, or short-segment adhesions	Treats underlying obstruction; avoids permanent shunt dependency	Re-adhesion, operative cord manipulation, limited applicability to extensive disease
Syringosubarachnoid shunt	Drain syrinx into subarachnoid space	Syrinx with patent receiving subarachnoid compartment	Direct decompression	Myelotomy; distal failure in scarred CSF space
Syringoperitoneal or syringopleural shunt	Drain syrinx outside spinal CSF space	Extensive scarring without reliable subarachnoid target	Does not require patent spinal receiving compartment	Shunt obstruction, infection, disconnection, dependency
Subarachnoid–subarachnoid bypass	Route CSF around obstruction	Dominant focal block with patent spaces above and below	Avoids myelotomy	Less effective with longer obstructions; does not relieve spinal cord tethering
Thecoperitoneal shunt	Divert thecal CSF and reduce pathological gradients	Extensive disease or impaired lumbosacral drainage	Avoids direct syrinx entry and extensive reconstruction	Limited long-term evidence of efficacy
Posterior fossa decompression	Restore craniocervical CSF communication	Foramen magnum or craniocervical arachnoiditis	Treats obstruction near fourth ventricular outlets	Recurrence and revision may occur
Flexible thecoscopy as an adjunct to open arachnolysis	Extend visualization and adhesiolysis through limited exposure	Multilevel adhesions accessible through endoscopic corridor	Reduces extent of open exposure	Small evidence base; uncertain durability; better at opening the obstructed CSF pathway than untethering

**Table 4:** Contemporary experimental models relevant to arachnoiditis-related syringomyelia [51,52,53,54,55].

Model	Experimental Approach	Major Finding	Translational Relevance	Principal Limitation
Ma et al., 2020 [53]	Chronic extradural spinal cord compression in rats	CSF-flow obstruction produced syringomyelia in 33/40 rats; decompression produced variable syrinx response	Demonstrates that sustained spinal CSF obstruction can generate syrinx formation	Does not reproduce intradural adhesive arachnoid fibrosis
Xu et al., 2021 [55]	Contusive spinal cord injury plus subarachnoid blood injection in rats	Produced cord tethering and chronically expanding extracanalicular cysts	Models interaction between spinal cord injury, subarachnoid pathology, tethering, and syrinx progression	Primarily a post-traumatic model rather than extensive inflammatory arachnoiditis
Johnson et al., 2023 [52]	Motorized spinal cord impact model	Perivascular spaces were enlarged in the perisyringeal spinal cord	Supports a potential role of perivascular fluid pathways in syrinx pathophysiology	Very small mechanistic study
Pukale et al., 2024 [54]	Quisqualic acid spinal injury plus subarachnoid kaolin in rats	Demonstrated increased perisyringeal osmolality and altered osmolytes/water transporters	Identifies molecular mechanisms that may contribute to syrinx expansion	Artificial inflammatory/chemical injury
Cui et al., 2025 [51]	Epidural-compression syringomyelia model followed by decompression	Persistent glial scarring limited syrinx regression despite restoration of subarachnoid CSF flow	Suggests that correction of CSF obstruction alone may not reverse established spinal cord pathology	Does not reproduce adhesive arachnoiditis itself

## Data Availability

No new data were created or analyzed in this study. Data sharing is not applicable to this article.

## Abbreviations

The following abbreviations are used in this manuscript:

CSF	Cerebrospinal fluid
CT	Computed tomography
MRI	Magnetic resonance imaging
